# Hill’s Stopping

**Published:** 1848-10

**Authors:** 


"HILL'S STOPPING."—Gutta Percha and bone ash, (phosphate of lime,) it seems, assume a somewhat costly shape when combined and put up in *small bottles*, and *labelled* "directions for use." Why, it is enough to put the advocates of amalgam into "*fits*," when an attempt is thus made to throw their favorite substance in the *shade!* And then, the way the *Inventor (?)* TRIES "to come the *big-figure* in his *laudable and praiseworthy efforts to pick up the dimes* is absolutely excruciating!

Spirits of Turpentine will be found an excellent local remedy for the gums, when there is a want of action in their capillaries, and a vitiated mucoid secretion of the lining membrane of the mouth; but, it cannot be used where "Hill's Stopping" has been introduced, because turpentine will dissolve it.

The enlightened dental profession must view with suspicion, if not with extreme disgust, every effort on the part of those practitioners who, by means of secret remedies, or "*patent penny whistles*," endeavor to construct fortunes by such mercenary practices as inflict shameful wrongs upon a noble profession, which they, unfortunately have disgraced, in the membership of beings so base. They have undoubtedly mistaken their calling; they should forthwith devote their valuable time, and eminent talents, to the more *appropriate* and *congenial* pursuits of peddling patent washing machines, wooden nutmegs, or white oak cucumber seeds.—ST. LOUIS ED.

